# Thickness Effect of 2195 Al–Li Alloy Friction Stir Weld Fracture Toughness

**DOI:** 10.3390/ma17153639

**Published:** 2024-07-23

**Authors:** Kejin Song, Peichen Liang, Xuesong Fu, Zhenggen Hu, Guoqing Chen, Wenlong Zhou

**Affiliations:** 1 School of Materials Science and Engineering, Dalian University of Technology, Dalian 116024, China; kjsong2000@163.com (K.S.); xsfu@dlut.edu.cn (X.F.); gqchen@dlut.edu.cn (G.C.); 2 Beijing Institute of Astronautical Systems Engineering, Beijing 100076, China; allenlkm@126.com (P.L.); firezhenggen@163.com (Z.H.)

**Keywords:** 2195 aluminum–lithium alloy, fracture toughness, friction stir welding, thickness effect, weld nugget zone

## Abstract

For damage tolerance design in engineering components, the fracture toughness value, *K_IC_*, of the material is essential. However, obtaining specimens of sufficient thickness from stir friction welded plates is challenging, and often, the experimental test values do not meet the necessary criteria, preventing the experimental fracture toughness, *K_q_*, from being recognized as plane strain fracture toughness *K_IC_*. The fracture toughness *K_q_* of 2195 Al–Li alloy welding seams with different thicknesses was measured on the forward and backward sides. Microstructure characterization was conducted by scanning electron microscope (SEM). The results indicated minimal significant differences in grain size between the advancing and retreating sides of the weld nugget zone. In specimens of the same thickness, fracture toughness measurements along the normal direction of the joint cross-section showed a high similarity between the advancing and retreating sides of the weld nugget zone. Utilizing the quantitative relationships between fracture toughness and sample thickness derived from both the fracture K and G criteria, it is possible to predict the fracture toughness of thick plates using thin plates. This study employs these relationships to calculate the fracture toughness *K_IC_* of 2195 aluminum–lithium alloy friction stir welds. The K_IC_ values obtained are 41.65 MPa·m^1/2^ from the fracture K criterion and 43.54 MPa·m^1/2^ from the fracture G criterion.

## 1. Introduction

As the manufacturing industry’s continuous demand for high performance and lightweight materials grows, the research and development as well as the application of aluminum–lithium alloys have increasingly garnered widespread attention [1]. Aluminum–lithium alloys, characterized by their low density, high strength and toughness, excellent fatigue resistance, and substantial elastic modulus, are ideally suited for environments demanding high strength and low weight. These alloys find widespread use in aerospace, transportation, military equipment, and other sectors. Currently, the 2195 aluminum–lithium alloy is predominantly used in the cryogenic fuel tanks of space shuttles and launch vehicles, replacing the 2219 aluminum–lithium alloy [2,3]. Fracture toughness, a critical performance indicator of engineering materials, refers to the critical stress intensity factor used in fracture mechanics to predict the residual strength of structures containing cracks. Accurate determination of fracture toughness is crucial for designing the damage tolerance of engineering components [4].

Generally, the measured value of fracture toughness increases with specimen thickness, and this relationship is influenced by factors including material properties, crack shape, and loading conditions [5,6]. This phenomenon occurs as the crack tip in a thin plate exists in a state of plane stress. With increasing plate thickness, the proportion of the crack tip undergoing plane strain also enlarges. When the fracture toughness attains a specific level, it commences to decrease. Upon the plate thickness reaching a particular value, the crack tip assumes a plane strain state. At this stage, the fracture toughness solely depends on the material properties.

In most countries, for the testing of plane strain fracture toughness *K_IC_*, the specimen thickness B must meet the requirement of $B\geq 2.5({\frac{K_{IC}}{\sigma_{s}})}^{2}$. If the specimen size fails to meet the validity criteria for *K_IC_*, the measured results can only be considered as plane stress fracture toughness *K_q_*, not as *K_IC_*. Since *K_q_* is affected by specimen size and is not an intrinsic property of the material, comparing the performance of different materials becomes problematic [7]. In engineering practice, directly measuring fracture toughness *K_IC_* is often impractical due to the difficulty in obtaining sufficiently thick specimens [8]. Consequently, researchers have extensively investigated the impact of specimen thickness on fracture toughness [9,10,11,12]. In 1991, Wallin [13] introduced the Master Curve approach to determine the fracture toughness of steel, which utilizes a single curve to describe the temperature dependency of steel’s fracture toughness and includes the corresponding expressions. Subsequently, numerous researchers have adopted the standard Master Curve method to determine the lower bounds of material fracture toughness [14,15,16]. Friction stir welding (FSW) is renowned as a welding method that produces joints with exceptional mechanical properties [17,18,19,20]. FSW joints typically exhibit high joint strength, closely matching the strength of the substrate [21]. The use of FSW technology for welding aluminum–lithium alloy sheets demonstrates significant engineering adaptability and promising application prospects [22,23,24]. However, bottlenecks remain in the process and implementation of FSW for large thick plates, making it extremely challenging to prepare test specimens that meet the *K_IC_* value criterion, and direct test results of *K_IC_* are unobtainable.

This study utilizes precise mathematical formulas to elucidate the relationship between fracture toughness and specimen thickness, guided by the fracture K criterion and fracture G criterion theories. This methodology is designed to determine the fracture toughness of materials of specific thicknesses using minimal experimental efforts. The relationship between fracture toughness and specimen thickness for 2195 Al–Li alloy FSW welds is established from the fracture toughness test data obtained from compact tensile specimens of varying thicknesses. Ultimately, the fracture toughness value *K_IC_* of the 2195 aluminum–lithium alloy friction stir weld nugget zone (NZ) in a plane strain state is accurately calculated.

## 2. The Basic Theory of the Relationship between Fracture Toughness and Specimen Thickness

In the framework of linear elastic fracture mechanics, the analysis of crack body fracture primarily focuses on fracture toughness K criteria and fracture toughness G criteria. The relationship between material fracture toughness and specimen thickness is established by integrating linear elastic fracture mechanics with the Mises yield criterion and Griffith’s theory. Jiyun Yang [25] proposed a theoretical formula for predicting the fracture toughness of plates with varying thicknesses by analyzing the stress field intensity near the crack tip using the plane strain method. Additionally, Hefei Li [8] developed a formula for calculating the fracture toughness of thick plates by considering the change in system energy during crack propagation.

Under linear elastic conditions:

(1) $$G_{IC}=\frac{K_{IC}^{2}}{E_{1}}\;(mode\;I\;fracture\;toughness)$$

(2) $$E_{1}=\frac{E}{1-v^{2}}\;(plane\;strain\;condition)$$

where *G* is the energy release rate, *E_1_* is the generalized elastic modulus, *K_IC_* is the mode I stress intensity factor for opening-type cracks, and v is Poisson’s ratio.

### 2.1. Fracture K Criterion: Relationship between Fracture Toughness and Specimen Thickness

According to Griffith’s surface energy theory, for materials experiencing plane strain conditions:

(3) $$K_{IC}=\sqrt{{2E}_{1}S}$$

where *S* is surface energy and *K_IC_* is the mode I fracture toughness.

According to the Barenblatt adsorption theory:

(4) $$S=\frac{1}{2}\int_{0}^{\delta^{*}}p(\delta )d\delta$$

where *δ* is the crack opening displacement in the vicinity of the crack tip adsorption zone, *p(δ)* is the adsorption force intensity on the upper and lower sides of the crack in the adsorption zone, and δ* is the limit value of crack opening displacement.

According to the Dugdale theory, where the plastic zone at the crack tip is regarded as an adsorption zone, the fracture toughness of the material under plane strain conditions can be expressed as:

(5) $$K_{C}=\frac{1}{2}\sqrt{E\int_{0}^{\delta^{*}}p\left(\delta \right)d\delta }$$

Given the plastic zone with slip planes inclined at a 45° angle to the plate plane, the crack opening displacement *δ* within this zone equals the product of the plastic zone normal strain *ε* and the plate thickness B, expressed as *δ* = B*ε*. Equation (3) can then be transformed as:

(6) $$K_{C}=\frac{1}{2}\sqrt{EB\int_{0}^{{Ɛ}^{*}}\sigma \left(Ɛ\right)dƐ}$$

when B→0, *K_q_* = α√B, the material is completely in a plane stress state; when B→∞, *K_q_* = *K_IC_*, the material is completely in a plane strain state.

(7) $${When\;B\in \left(0,\infty \right),K}_{q}={\alpha B}^{1/2}e^{-\beta B^{m}}+K_{IC}\left(1-e^{-\beta B^{m}}\right)$$

where α and β are material constants and m is the exponent constant.

Utilizing the fracture toughness *K_q_* values from specimens with various thicknesses, the material constants α and β can be optimized through fitting using the least squares method. This facilitates the determination of the fracture toughness *K_IC_* of the material in a plane strain state (B = ∞).

### 2.2. Fracture G Criterion: Relationship between Fracture Toughness and Specimen Thickness

Based on the assumptions that: The effect of specimen thickness on the ratio of tensile fracture zone to shear fracture zone is relatively negligible for the material. The energy release rate criterion must be adhered to during crack propagation. As the crack advances to the critical unstable state, the total energy expenditure can be partitioned into that consumed by the tensile fracture zone and that consumed by the shear fracture zone, which can be expressed by Equation (8):

(8) $$U=U_{C}+U_{S}$$

where *U* is the total energy consumed when the crack extends to the critical unstable state, *U_C_* is energy consumed in the tensile fracture zone, and *U_S_* is energy consumed in the shear fracture zone.

**Figure 1:**
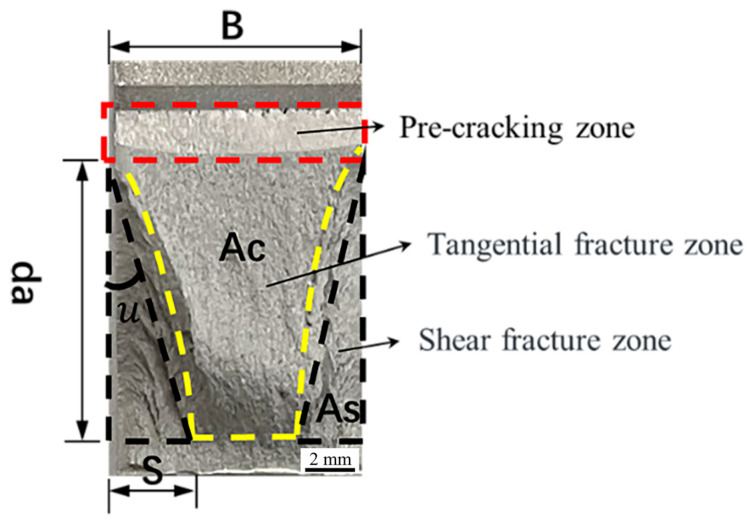
Fracture surface at critical unstable state of a crack.

During the crack propagation phase, the energy release rate G does not exhibit a uniform distribution. It reaches its maximum in the central region and decreases gradually as it approaches the specimen’s surface [26]. Consequently, the crack propagation rate in the tensile region exceeds that in the shear zone [27]. As the crack propagates within the tensile fracture zone, plastic deformation arises on the specimen surface [28], and the energy expended on plastic deformation can be regarded as the energy spent within the shear fracture zone. Both the shear fracture zone and the tensile fracture zone within the macroscopic fracture morphology are treated as geometrically regular shapes, as depicted in Figure 1. However, in cases where the specimen thickness is excessively small, the macroscopic fracture characteristics no longer exhibit a distinct tensile fracture zone, leaving only the shear fracture zone.

(9) $$A_{S}=Sda$$

(10) $$A_{C}=(B-S)da$$

(11) $$A_{T}=Bda$$

(12) S=uda

where *S* is the shear lip width, da is the distance when the crack begins to extend to the critical unstable state, μ is the slope angle θ between the crack tip and the maximum shear lip width, *A_T_* is the area of the entire crack propagation stage, *A_C_* is the area of the tensile fracture zone, and *A_S_* is the area of the shear fracture zone.

According to the principle of energy conservation, Equation (8) can be formulated as:

(13) $$G_{q}A_{T}=G_{C}A_{C}+G_{S}A_{S}$$

where *G_Q_* is energy consumption per unit distance of crack propagation on the entire fracture surface, *G_C_* is energy consumption per unit distance of crack propagation in the tensile fracture zone, and *G_S_* is energy consumption per unit distance of crack propagation in the shear fracture zone.

Substituting Equation (9) through Equation (12) into Equation (13), we derive:

(14) $$BG_{C}+\mu \left(G_{S}-G_{C}\right)da=BG_{q}$$

Additionally, given that the specimen thickness minimally impacts the angle between the crack tip and the shear lip, and *G_IC_* relies on the crack length and local stress [29], *μ* and *da* can be regarded as constants. Consequently, Equation (14) can be formulated as:

(15) $$\mu \left(G_{S}-G_{C}\right)da=C$$

where *C* is a constant.

Combining Equations (1), (14) and (15), we obtain:

(16) $$BK_{q}^{2}=BK_{IC}^{2}+C_{0}$$

## 3. Materials and Methods

### 3.1. Materials and Manufacturing Process

**Figure 2:**
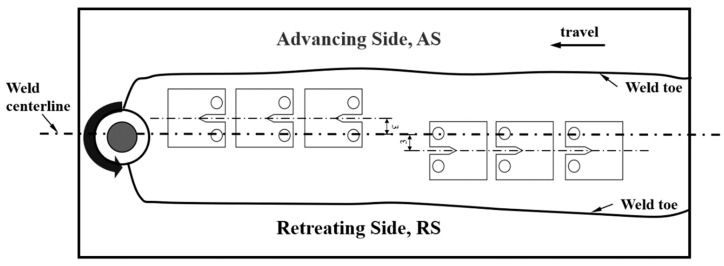
Schematic diagram of welding joints and fracture toughness test specimens.

This study utilizes a 12 mm thick 2195 aluminum–lithium alloy plate, with its material composition detailed in Table 1. The selected optimal welding parameters were the rotation speed of 550 r/min and welding speed of 100 mm/min. Compact tensile specimens (CT) with thicknesses of 6 mm, 8 mm, and 10 mm were extracted from the friction stir welding welds of the plates, as depicted in Figure 2. This paper primarily investigates the fracture toughness of both the advancing side (AS) and the retreating side (RS) of the weld nugget zone in friction stir welding. To mitigate the impact of sampling at different depths within the weld nugget zone on the test results, specimens were randomly collected from both the top and bottom of the weld nugget zone, ensuring the notch midline of each specimen is 3 mm from the weld’s center line.

### 3.2. Experiment Preparation

The polished specimen was etched using Keller’s reagent (10 mL HNO_3_ + 6 mL HCl + 4 mL HF+ 190 mL H_2_O), and its microstructure was subsequently analyzed using an optical microscope (OM, AxiScope·5). EBSD samples were electropolished using a mixture of 10% HClO_4_ and 90% C_2_H_5_OH at 16 V and −15 ° C for 30 s. EBSD analysis was performed using a scanning electron microscope (SEM, JSM-IT800) at an operating voltage of 20 kV and a scanning step size of 0.6 μm or 1.5 μm. Prior to testing, the fracture toughness specimens were ultrasonically cleaned with anhydrous ethanol and stored in a dry, oxidation-free environment. The fracture toughness test was conducted on an MTS Landmark fatigue testing machine, and the crack length at the fracture site was measured using an extensometer. The load was determined based on the yield strength (*σ*_*s*_) of the 2195 Al–Li alloy longitudinal plate, set at 500 MPa·m^1/2^, with the actual load applied being 0.8*σ*_*s*_. For the 6 mm, 8 mm, and 10 mm specimens, three data sets were tested under each material condition.

## 4. Results and Discussion

### 4.1. Microstructure Analysis

#### Grain Feature

**Figure 3:**
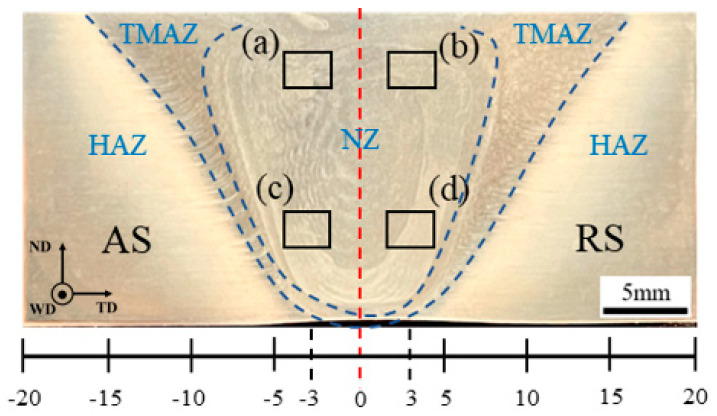
Macroscopic morphology of cross section of 2195 Al–Li alloy friction stir welding joint and metallographic sampling position: (**a**) NZ (Top, AS); (**b**) NZ (Top, RS); (**c**) NZ (Bottom, AS); (**d**) NZ (Bottom, RS).

Figure 3 displays the macroscopic morphology and microstructure of the typical welded joint area. The friction stir weld is in good condition, free of macroscopic cracks and voids. WD, ND, and TD denote the welding mixing head’s travel direction, the mixing head’s normal direction, and the weld’s transverse direction, respectively. Owing to variations in material flow during welding, the weld zone exhibits the characteristic “onion ring” morphology of FSW [30]. During friction stir welding, the weld nugget zone’s width at the top is greater than at the bottom. Consequently, this study positions the machining notch’s center line of the compact tensile specimen 3 mm from the weld’s center line, ensuring consistent sampling from both the top and bottom of the weld nugget zone.

**Figure 4:**
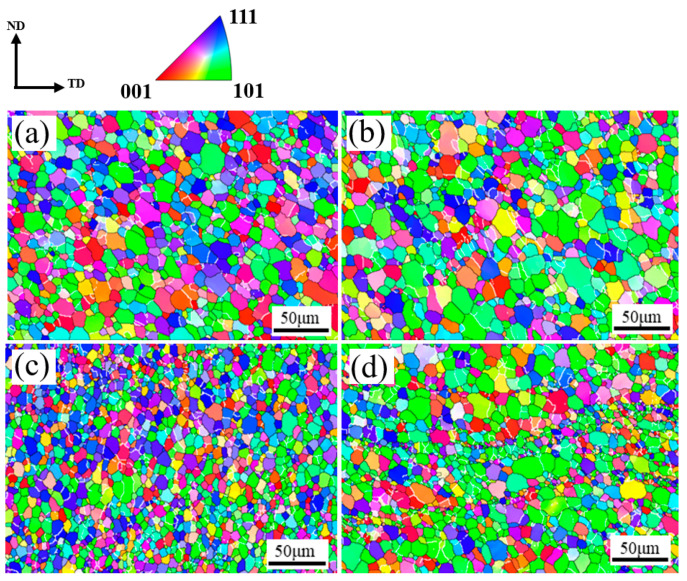
Crystallographic orientations at various marked positions in the microstructures: (**a**) NZ (Top, AS); (**b**) NZ (Top, RS); (**c**) NZ (Bottom, AS); (**d**) NZ (Bottom, RS).

During the welding process, the friction between the stirring tool and the plate generates heat, causing violent and complex deformation of the material in the weld nugget zone and resulting in dynamic recrystallization. Consequently, the weld nugget predominantly comprises equiaxed grains, characterized by marked dynamic recrystallization features. The grains at the top of the weld nugget zone are influenced by the adjacent shoulder of the mixing tool, receiving more heat input [31]. The energy stored within the grains promotes their growth. Compared to the top, the heat input at the bottom is lower; consequently, due to the bottom material and the bottom plate, heat conduction and temperature cooling rates are faster. The energy within the grains facilitates recrystallization but does not reach the threshold required for grain growth. Consequently, the grain size at the bottom is significantly smaller than at the top. Because the material flow on the AS of the weld aligns with the direction of the stirring head, there is increased friction between the material and the tool, resulting in greater friction heat generation. This heat promotes grain growth under high temperatures. In contrast, the RS experiences less friction and heat, inhibiting grain growth. Consequently, low angle boundaries (LABs) with misorientation angles between 2° and 15° are illustrated by white lines, while high angle boundaries (HABs) with misorientation angles above 15◦ are noted by black lines in this paper. As shown in Figure 4, the grain size on the AS of the weld nugget zone is slightly larger than that on the RS [32].

**Figure 5:**
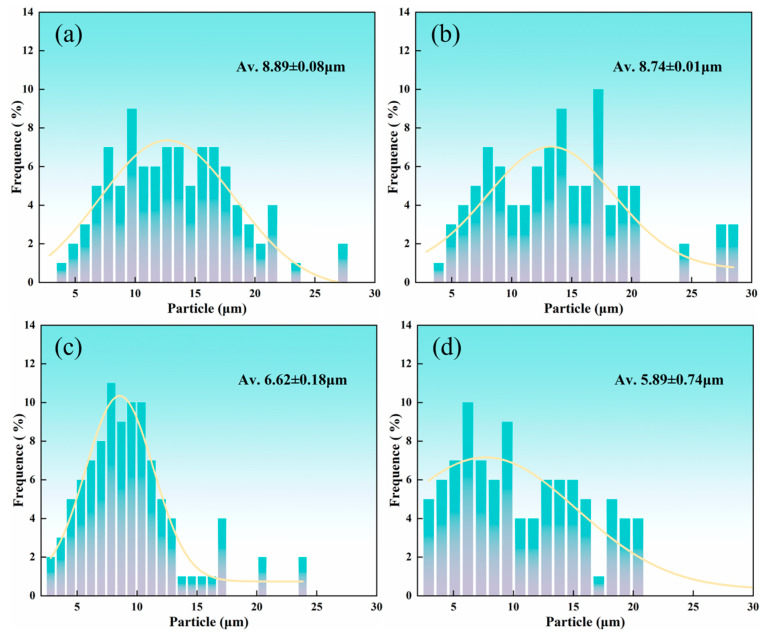
Grain size of different regions (NZ) in welding joints: (**a**) NZ (Top, AS); (**b**) NZ (Top, RS); (**c**) NZ (Bottom, AS); (**d**) NZ (Bottom, RS).

Under normal conditions, fine equiaxed grains can limit crack propagation and enhance the fracture toughness of materials. The average grain size at the top of the AS of the weld nugget zone, as calculated by Aztec Crystal software version 2.1, is 8.89 ± 0.08 μm, while at the bottom it measures 8.74 ± 0.01 μm. On the RS, the average grain size at the top is 6.62 ± 0.18 μm and at the bottom is 5.89 ± 0.74 μm, as shown in Figure 5.

### 4.2. Fracture Toughness Test Results of Different Thicknesses

**Figure 6:**
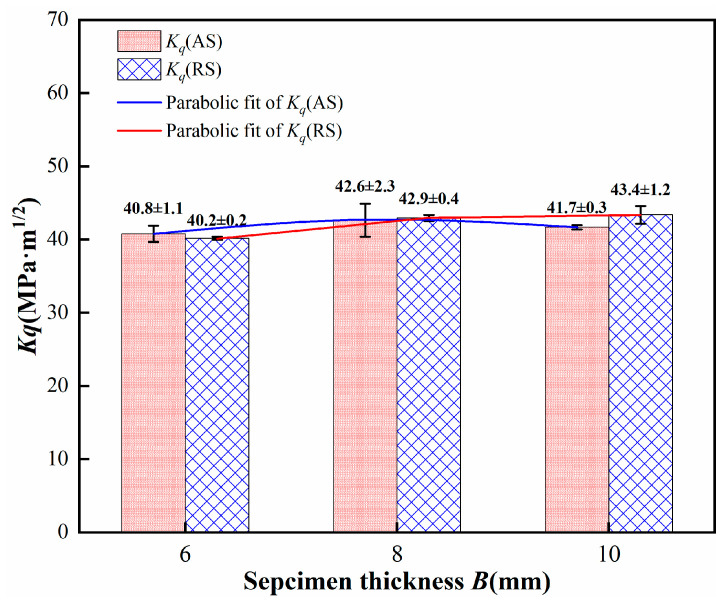
Fracture toughness diagram of specimens with different thicknesses for the AS and RS of welded joints.

The fracture toughness of different thickness specimens of 2195 aluminum–lithium alloy friction stir welded joints was tested. Table 2 presents the specimen dimensions and test data. The results revealed that for specimens of the same thickness (6–8 mm), the difference in fracture toughness values between the AS and the RS ranged from 1.5%, 0.7%, to 4.1%, with grain size in the weld nugget zone having minimal impact on fracture toughness. For varying thicknesses, the fracture toughness of the AS specimens first increases with thickness before decreasing, while that of the RS continuously increases, as shown in Figure 6. The difference in fracture toughness values between the AS and the RS for the same thickness is minimal, indicating that fracture toughness is primarily related to specimen thickness. To reduce experimental error and enhance the precision of subsequent fits between fracture toughness and specimen thickness, this study disregards the effects of the AS and the RS of the weld nugget zone. The truncation method was used to eliminate significant deviations in test data for similar specimens. Four optimal data points from each group were selected to calculate the average fracture toughness for thickness specimens, used to fit the relationship between the fracture toughness of the friction stir weld joints and specimen thickness. The resulting average fracture toughness values for specimens of different thicknesses were: 40.2 ± 0.2 MPa·m^1/2^ for 6 mm, 42.7 ± 0.7 MPa·m^1/2^ for 8 mm, and 42.2 ± 0.7 MPa·m^1/2^ for 10 mm.

### 4.3. Modeling the Relationship between Fracture Toughness and Specimen Thickness

**Figure 7:**
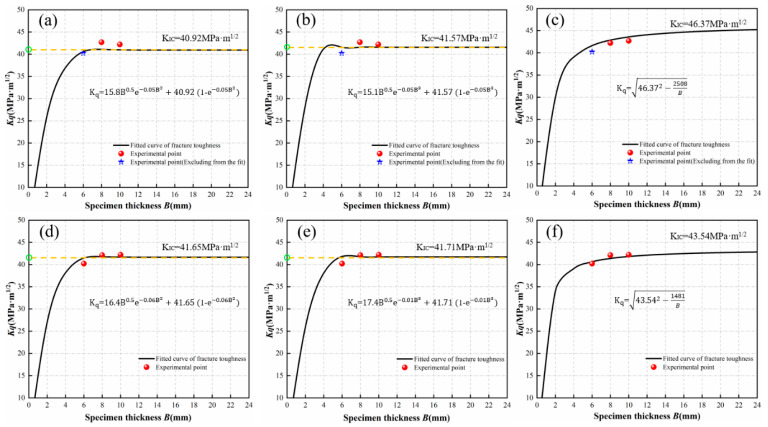
Curve of fracture toughness variation with thickness: (**a**–**c**) fit using two sets of experimental points; (**d**–**f**) fit using three sets of experimental points, and in addition: (**a**,**d**) curve fitted by K criterion (m = 2); (**b**,**e**) curve fitted by K criterion (m = 3); and (**c**,**f**) curve fitted by G criterion.

Using the data from Table 2, fracture toughness was modeled in relation to specimen thickness using theoretical formulas based on the “Fracture K Criterion” and “Fracture G Criterion.” To investigate the impact of the number of data points on the accuracy of the fitted curves, this study used two sets of experimental data points for 6 mm and 8 mm, and three sets for 6 mm, 8 mm, and 10 mm. The data points for 6 mm and 8 mm thicknesses were input into Equation (7), fitted using the least squares method. To improve the fit’s goodness, the exponent constant B^m^ in Equation (7) was optimized, resulting in Formulas (17) and (18) for the relationship between fracture toughness and specimen thickness under the effect of thickness, with exponent constants of 2 and 3, respectively. Data points for 6 mm and 8 mm thicknesses were also fitted using Equation (16), resulting in Equation (19) for the relationship under thickness effects. Similarly, data points for 6 mm, 8 mm, and 10 mm thicknesses were fitted using Equations (7) and (16), producing the relationships shown in Equations (20)–(22). The resulting curves of specimen thickness versus fracture toughness are displayed in Figure 7.

(17) $$\;K_{q}=15.8B^{0.5}e^{-{0.05B}^{2}}+40.92\left(1-e^{-{0.05B}^{2}}\right),$$

(18) $$\;K_{q}=15.1B^{0.5}e^{-{0.05B}^{3}}+41.57(1-e^{-{0.05B}^{3}})$$

(19) $$\;K_{q}=\sqrt{\;46.37^{2}-\frac{2508}{B}}$$

(20) $$\;K_{q}=16.4B^{0.5}e^{-{0.06B}^{2}}+41.65(1-e^{-{0.06B}^{2}})$$

(21) $$\;\;K_{q}=17.4B^{0.5}e^{-{0.01B}^{3}}+41.71({1-e}^{-{0.01B}^{3}})$$

(22) $$K_{q}=\sqrt{43.54^{2}-\frac{1481}{B}}$$

For the fracture K criterion, a larger number of data points results in lower fitted *K_IC_* values, and the fitting curve with a wall thickness exponent of m = 2 exhibits a relative error that is 1.5% greater than that with an exponent of m = 3. Regarding the fracture G criterion, Equation (16) shows that *K_q_*^2^ and *K_IC_*^2^ are linearly and positively correlated; more data points lead to a *K_IC_* value closer to the true value. The relative error in *K_IC_* values fitted from two and three sets of data points reaches 6.1%, as shown in Table 3.

### 4.4. Error Analysis and Test Verification of Fitting Relation

The discrepancy between the experimental results and the calculated values of fracture toughness is presented in Table 4. The number of test data points for different thicknesses influences the relative error of the fitting results; therefore, a higher number of data points results in a smaller relative error. When applying the fracture K criterion to fit the curve, the accuracy of the theoretical curve can be enhanced by adjusting the power of the polynomial with respect to plate thickness B. In this study, three test data points were employed to align the curve more closely with the overall test results, and the overall relative error for the power m = 2 of plate thickness B is further reduced compared to the power m = 3. When using the fracture G criterion for curve fitting, having a greater number of test data points across different thicknesses generally results in a smaller relative error and a better curve fit.

Therefore, both theoretical models are capable of describing the fracture toughness of welded joints in the 2195 Al–Li alloy across various thicknesses, and can be utilized to determine the fracture toughness of materials of a specific thickness. However, for the fracture G criterion, the macro fracture characteristics of the specimens must include both a positive fault zone and a shear fracture zone. If the specimen thickness is too small, the characteristics of the positive fault zone disappear, leaving only the shear fracture zone. This situation does not fulfill the conditions for small yield and thus has certain limitations. When deriving fracture toughness values for other thicknesses using these theoretical models, the choice should be based on the actual situation. Incorporating more test data points will help to fit a relationship curve between fracture toughness and thickness that more accurately reflects the material properties. To minimize errors, three distinct test data points regarding thickness were selected to fit the curve equation. The curve equation was fitted based on the K criterion. With the power m = 2 of plate thickness B, the fracture toughness value was determined as 41.65 MPa·m^1/2^. Based on the curve equation fitted using the G criterion, the fracture toughness value obtained was 43.54 MPa·m^1/2^.

## 5. Conclusions

(1) In 2195 aluminum–lithium alloy friction stir welding, the grain size on the advancing side of the weld nugget zone is slightly larger than on the retreating side, and the grains at the bottom of the weld nugget zone are smaller than those at the top. At the top of the AS, the average grain size measures 8.89 ± 0.08 μm, compared to 8.74 ± 0.01 μm at the bottom. On the RS, the average grain size is 6.62 ± 0.18 μm at the top and 5.89 ± 0.74 μm at the bottom.
(2) Utilizing the fracture toughness criteria K and G, a relational formula linking the fracture toughness of 2195 aluminum–lithium alloy friction stir welds to specimen thickness was derived. The plane strain fracture toughness values were computed using this derived formula. There is a negligible difference in the fracture toughness *K_IC_* values between the advancing and retreating sides of the 2195 aluminum–lithium alloy friction stir welds. To reduce fitting errors, the final *K_IC_* values, derived from the fracture K criterion, are 41.65 MPa·m^1/2^, while those from the fracture G criterion are 43.54 MPa·m^1/2^.

## Figures and Tables

**Table 1:** Chemical composition of 2195 aluminum–lithium alloy (wt%).

Cu	Li	Zr	Mg	Ag	Ti	Si	Fe	Al
3.84	0.87	0.09	0.34	0.31	0.08	0.12	0.11	Balance

**Table 2:** Fracture toughness test data for 2195 aluminum–lithium alloy friction stir welded joint specimens of different thicknesses.

Number	Specimen Width W (mm)	Specimen Thickness B (mm)	Av. Crack Length a (mm)	K_q_ (MPa·m^1/2^)	Note	Av. K_q_ (MPa·m^1/2^)
AS(6)-1	30.1	6.0	14.7	40.4		40.2 ± 0.2
AS(6)-2	30.2	6.0	14.9	39.9	×	
AS(6)-3	30.1	5.8	14.9	42	×	
RS(6)-1	30.1	5.8	15.4	40		
RS(6)-2	30.3	6.0	14.4	40.1		
RS(6)-3	30.3	5.9	14.9	40.4		
AS(8)-1	30.0	8.1	14.8	41.8		42.7 ± 0.7
AS(8)-2	30.1	8.0	16.1	45.2	×	
AS(8)-3	30.0	8.0	14.8	40.9	×	
RS(8)-1	29.8	8.0	14.4	43		
RS(8)-2	30.2	8.1	14.1	43.3		
RS(8)-3	29.9	8.0	13.9	42.5		
AS(10)-1	29.7	10.1	15.3	41.5	×	42.2 ± 0.6
AS(10)-2	29.9	10.2	15.0	42		
AS(10)-3	29.8	10.0	15.4	41.5		
RS(10)-1	30.4	10.2	14.9	42.4		
RS(10)-2	30.3	10.1	15.0	44.7	×	
RS(10)-3	30.2	10.1	14.7	43		

Note: To enhance data precision, the truncation method was used to process the data; an “×” indicates data points that were discarded.

**Table 3:** Relative errors of fit results for different numbers of experimental data points.

Numbers	K_q_ (MPa·m^1/2^) K Criterion (m = 2)	Relative Error	K_q_ (MPa·m^1/2^) K Criterion (m = 3)	Relative Error	K_q_ (MPa·m^1/2^) G Criterion	Relative Error
2	40.92	1.8%	41.57	0.3%	46.37	6.1%
3	41.65		41.71		43.54	

**Table 4:** Relative error of fitting different experimental data points.

Number of Experimental Points	B (mm)	K Criterion (m = 2)	K Criterion (m = 3)	G Criterion
2	6	0.9%	3.3%	3.4%
	8	4.0%	2.7%	0.4%
	10	3.0%	1.5%	3.2%
3	6	3.0%	3.8%	1.0%
	8	2.2%	2.3%	3.2%
	10	1.2%	1.2%	0.9%

## Data Availability

The original contributions presented in the study are included in the article, further inquiries can be directed to the corresponding author.
